# Male axillary accessory breast cancer: A case report

**DOI:** 10.1097/MD.0000000000019506

**Published:** 2020-03-13

**Authors:** Minglei Bi, Danyi Li, Yipeng Su, Pengfei Sun, Yan Gao

**Affiliations:** a Department of Plastic Surgery, The Affiliated Hospital of Qingdao University; b General Surgery, Qingdao West Coast New Area Central Hospital, Qingdao, China.

**Keywords:** accessory breast cancer, accessory mammary gland, axilla, male

## Abstract

**Rationale:**

Accessory breast cancer is extremely rare among all cancerous diseases, especially in male patients. There were only few male axillary accessory breast cancer cases that have been reported in scientific literatures so far. Hereby, we would like to discuss a case of male axillary accessory breast cancer found in our hospital.

**Patient concerns:**

We report a male senile patient suffering from a painful, enlarged, and hardened right axillary mass for more than 20 years. He came for further treatments due to progressive growth of the mass for 11 months with bloody ulceration for more than 1 month.

**Diagnosis:**

Pathological examination manifested a grade II infiltrating ductal carcinoma derived from the accessory mammary gland (right axilla), with invasion of local skin. Immunohistochemical examination result: estrogen receptor (++) 90%, progesterone receptor (+++) 100%, human epidermal growth factor receptor-2 (1+), ki67 (20% positive), prostate specific antigen (−), caudal-related homeobox-2 (−), thyroid transcription factor-1 (−), Synaptophysin (+), NapsinA (1), and CK7 (−).

**Interventions:**

Modified radical mastectomy and axillary lymph nodes clearance were performed on the accessary breast cancer under general anesthesia. Postoperatively, endocrine therapy was provided for the patient, orally-taken Letrozole was recommended for the rest of the patient's life.

**Outcomes:**

The patient recovered uneventfully and was discharged 3 days after the operation. The patient continued to take Letrozole orally regularly at home and no signs of recurrence were observed.

**Conclusion:**

Axillary accessory breast cancer in males is extremely rare, with no conspicuous and typical clinical presentations, which leads to inevitable neglect by clinicians. Therefore, there is significant necessity for clinicians to be cautious with this type of disease.

## 1 Introduction

Accessory mammary glands, also known as aberrant mammary glands, which characterize the disease of polymastia, may also be associated with polythelia.^[1]^ There are 6 to 8 pairs of breast primordium distributed on the lines bilaterally from axillaries to inguinal glands on human bodies.^[2]^ In the embryonic stage, all pairs of primordium are supposed to gradually degenerate except for the one distributed on the chest. A certain percentage of the population has superfluous mammary glands which do not degenerate or degenerate incompletely, manifested as the accessory mammary glands. The incidence of this disease is 1% to 6% in general population and the ratio of male to female is about 1:5.^[3]^ Although glandular diseases can also develop in accessory mammary glands, cases of malignant tumors occurring in the accessory mammary glands are extremely rare, especially in male patients.^[4]^ Therefore, clinicians are short of clinical experience of diagnosis and treatment for male accessory breast cancer, so that the aim of this article is to help clinicians better understand this type of disease.

## 2 Case presentation

**Figure 1:**
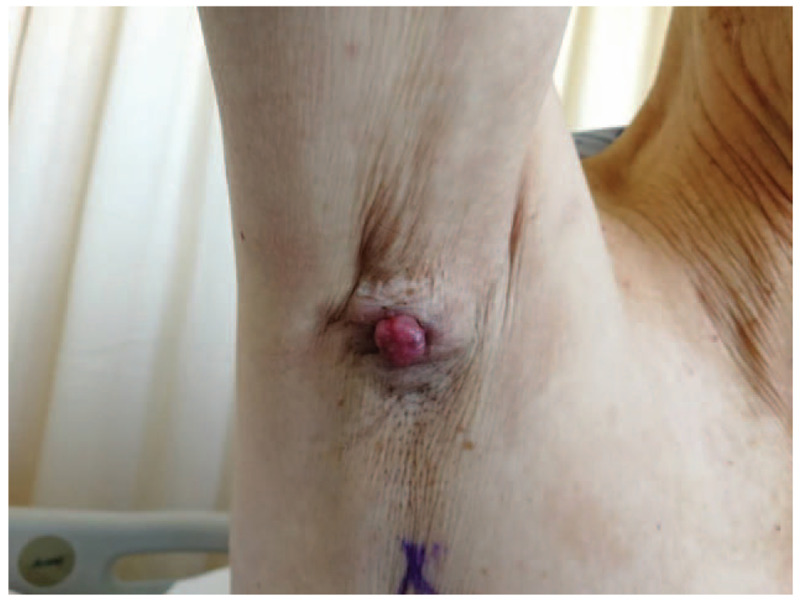
The cauliflower-like mass on the right axilla, which is 2.5 cm × 2.4 cm in size, 1.0 cm above the skin surface. The boundary is unclear, the shape is irregular, and the mass is immovable.

An 84-year-old male patient had a mass in his right axilla for more than 20 years. He came for further treatment due to its progressive growth for 11 months with bloody ulceration for more than 1 month. The initial size of the mass was 1.0 cm × 1.5 cm × 1.0 cm, similar to the size of a piece of peanut. It was hard and immovable. The boundary between tumor and normal skin was unclear. About 11 months ago, the patient felt the mass was increasing rapidly, expanding to 2.5 cm × 2.4 cm × 1.8 cm in size, and raised 1.0 cm above the skin surface (Fig. 1). Besides, the pain when touched gradually became conspicuous. One month ago, the mass started to swell and ulcerate. No signs of enlargement of bilateral supraclavicular lymph nodes were found.

**Figure 2:**
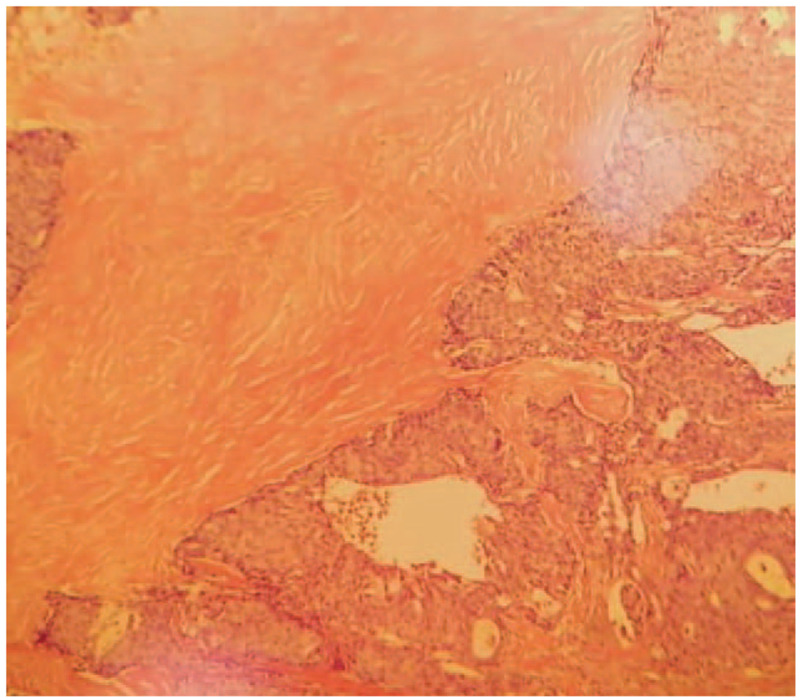
Pathological examination result (HE ×100) and pathological examination result (HE ×400). The resected tissue showed invasive growth. The tumor cell nuclei are deep stained and characterized by heteromorphism and caryocinesia. HE = hematoxylin and eosin.

**Figure 3:**
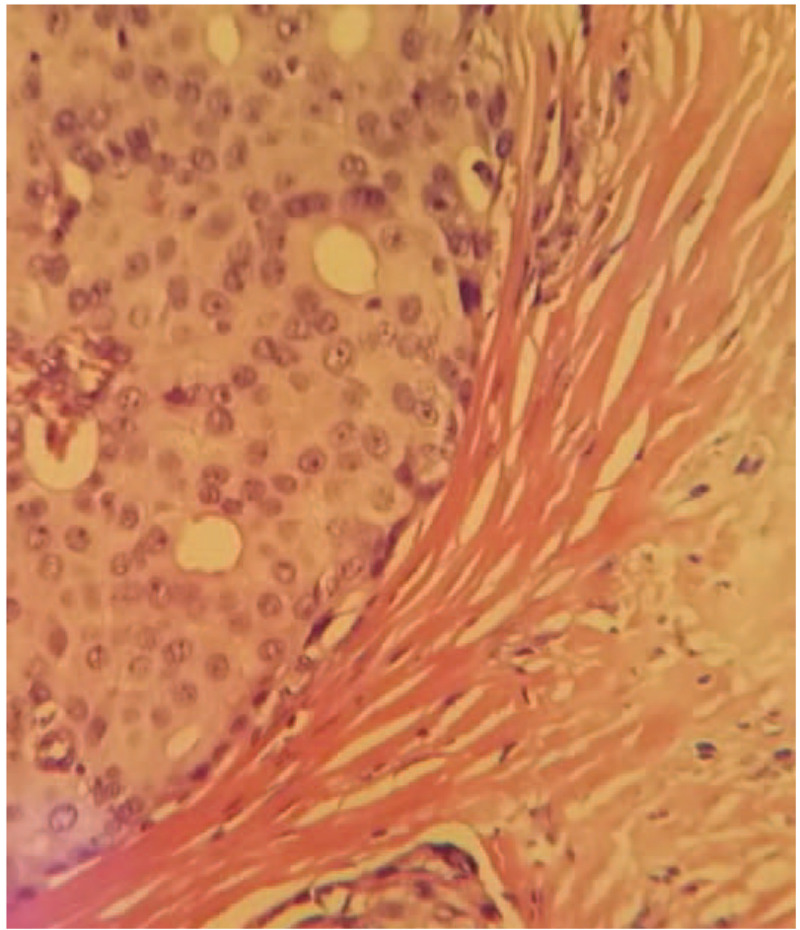
Pathological examination result (HE ×100) and pathological examination result (HE ×400). The resected tissue showed invasive growth. The tumor cell nuclei are deep stained and characterized by heteromorphism and caryocinesia. HE = hematoxylin and eosin.

Auxiliary examination:

1. Positron emission computed tomography showed the following:
  (1) A nodule of the density of subcutaneous soft tissue in the right axilla, the boundary was unclear, some layers protruded to the skin surface, with increase of metabolism. The Standardized Uptake Value max level was 8.8, considered malignant tumor.
  (2) Two enlarged lymph nodes in the right axilla, with abnormal increase of metabolism. The Standardized Uptake Value max level was 13.0, considered lymph node metastasis.
  (3) No significant signs of malignant primary tumor in other parts of the body.
2. Pathological Test: Grade II infiltrating ductal carcinoma derived from the accessory mammary gland (right axilla) with invasion of local skin.
3. Immunohistochemical examination result: estrogen receptor (++) 90%, progesterone receptor (+++) 100%, human epidermal growth factor receptor-2 (1+), ki67 (20% positive), prostate specific antigen (−), caudal-related homeobox-2 (−), thyroid transcription factor-1 (−), synaptophysin (+), NapsinA (1), and CK7 (−) (Figs. 2 and 3).

**Figure 4:**
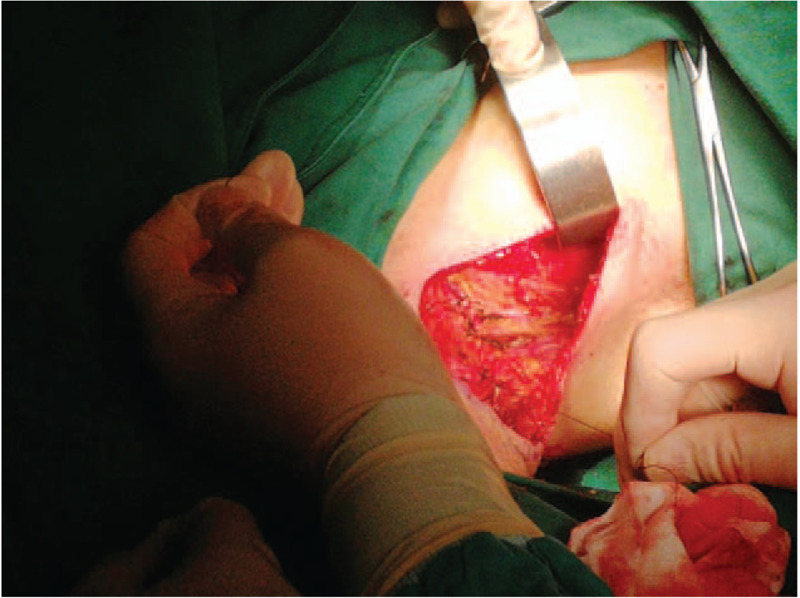
The operating surgeon removes the lymph nodes sequentially and arrests the bleeding thoroughly.

**Figure 5:**
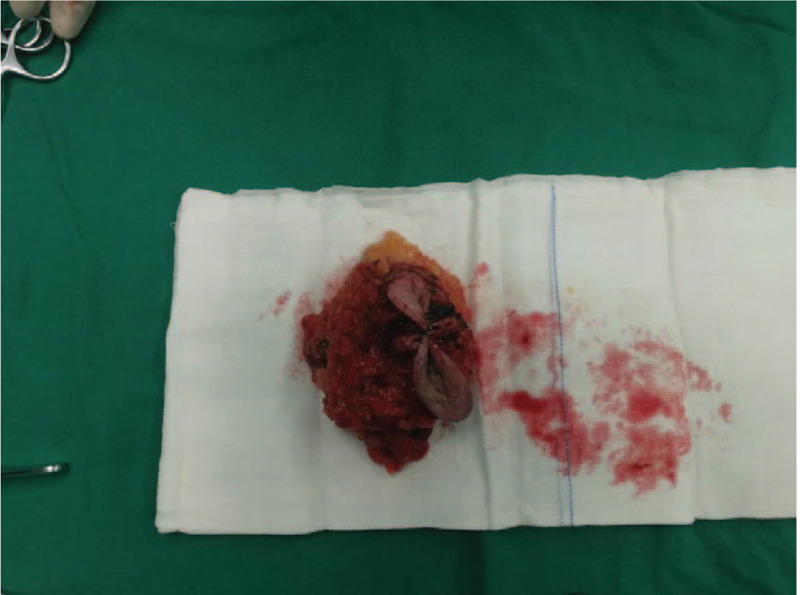
The operating surgeon removes the cauliflower-like mass in the right axilla and the accessory mammary gland completely.

After exclusion of operative contraindications, we decided to perform radical mastectomy of the accessory mammary gland and adjacent lymph nodes clearance under general anesthesia. First, we performed a spindle-shaped incision which was 3.0 cm around the mass. Second, the accessory mammary gland and the spindle-shaped skin flap were integrally dissociated by electronic knife. Thirdly, interpectoral lymph nodes, lateral pectoralis minor lymph nodes, anterior latissimus dorsi lymph nodes, axillary lymph nodes, posterior pectoralis minor lymph nodes were removed successively. Then, we removed the specimen after ligating and cutting off the arteries, veins and lymph glands. During the operation, we protected long thoracic nerve and dorsal thoracic vascular nerve. The operational region was irrigated with warm water (36°C–37°C), and the bleeding was arrested thoroughly. Parasternal and axillary drainage tubes were placed respectively. Finally, the operational region was packed with pressure bandage for at least 5 days (Figs. 4 and 5).

According to the Guidelines of Treatments for Breast Diseases in 2017, we decided to provide this patient with only endocrine therapy postoperatively. We recommended that the patient should take Letrozole orally for the rest of his life.

The patient recovered uneventfully and was discharged 3 days postoperatively, with no significant pain, hematoma, infection, or any functional disorder during upper limb activities. The patient was followed up regularly every 3 to 6 months, no signs of recurrence were observed. (Outcomes and follow-up).

## 3 Discussion

The predisposing occurring site of accessory breast cancer is axilla, followed by groin. The age of onset is above 50 years old.^[5–6]^ Patients often come for further treatments with the presentation of a palpable and painful mass in the axilla. Female patients often feel discomfort in axilla, which is related to menstrual cycle. The tumor is usually located in the superficial layer of the axilla and sometimes protrudes to the skin surface. If the skin is invaded by the tumor, it becomes red, swollen and shows the “orange peel” appearance.^[7]^ The texture of the tumor is tough and becomes hard gradually. Its shape is irregular and its boundary is unclear. Besides, the tumor is immovable and painful especially when being touched. Axillary accessory breast cancer is often accompanied by swelling and hard enlargement of the ipsilateral axillary or supraclavicular lymph nodes. These lymph nodes can fuse with each other occasionally. Accessory breast cancer developed in the groin is often accompanied by the enlargement of ipsilateral inguinal lymph nodes, sometimes with fusion.^[8]^ In this article, the reported case is a senile male patient, clinical presentations of aged population are often atypical due to atrophy of the accessory mammary gland.

This disease should be distinguished from the following diseases:

1. Axillary or inguinal lymphadenitis,^[9]^ mostly caused by infection of staphylococcus aureus and hemolytic streptococcus. Acute lymphadenitis is characterized by acute inflammation, with symptoms of redness, swelling, heat, pain, and so on. These symptoms can be alleviated by timely and regular use of anti-biotic and anti-inflammatory treatments. Chronic lymphadenitis is characterized by its long-term course and mild symptoms. The lymph nodes are movable and the painless.
2. Axillary or inguinal lymph node tuberculosis, also called scrofula in traditional Chinese medicine (TCM), of which the pathogen is mycobacterium tuberculosis.^[10,11]^ Initially, enlarged lymph nodes are hard, painless and movable. The course of this disease continues to develop and perilymphadenitis may occur. In its late stage, the lymph nodes start to develop cheesy necrosis, liquefaction and cold abscesses. The skin can ulcerate, which is also called “mouse sores” in TCM. A minority of patients suffer from systemic symptoms such as low fever, night sweat, and loss of appetite.
3. Breast cancer of axillary caudal region from the ipsilateral side. It belongs to the category of breast cancer, with extension to the axilla. The clinical manifestations are similar to those of accessory breast cancer.^[12]^ Only experienced radiologists can differentiate this disease from accessary breast cancer through ultrasound, molybdenum target radiography and magnetic resonance imaging (MRI). Pathological examination is the golden standard for diagnosis.
4. Lymph node metastasis^[13]^: ipsilateral axillary lymph node is the most common site of metastasis in breast cancer, about 40% of breast cancer patients are found to have axillary lymph node metastasis.^[14]^ However, what needs to be specified is that ipsilateral axillary lymph node metastasis can also occur in accessory breast cancer, the reported case in this article is a typical example. Therefore, the key point of differentiation for this disease is to exclude malignant tumors which may cause axillary lymph node metastasis in other parts of the body, especially for breast cancer on the same side.
5. Skin-derived tumors^[15–18]^:
  (1) Apocrine gland carcinoma. Its tumor cells are large and Periodic Acid-Schiff staining positive;
  (2) Basal cell carcinoma, which is often caused by over-exposure to sunlight. Most of them are superficial lesions, the development of disease is slow. Initially, circular spots of bright surface with pearl-like bulge edges can be found, generally are without inflammatory reaction. In the late stage, the lesion can ulcerate^[19,20]^;
  (3) Squamous cell carcinoma, which is mainly relevant to factors such as ultraviolet, chemical reagent and scarring, and so on. The earliest appearance is infiltrating hard spot, then the size of the skin lesion increases rapidly.^[21]^ In the late stage, the center of the skin lesion ruptures and ulcerates;
  (4) Sebaceous adenoma, a benign tumor of which the diameter is normally 1 to 2 mm, the color is yellow or fleshy, can occur in lesions of seborrheic keratosis or sebaceous nevus, or maybe a kind of manifestations of Torre–Muir syndrome.^[22]^

The auxiliary examinations of accessory breast cancer have not been standardized up to date. The diagnosis mainly relies on colorful ultrasound, MRI, molybdenum target radiography and pathological examination of the superficial mass. Colorful ultrasound, MRI and molybdenum target radiography can objectively manifest the shape and location of the tumor, thus are of great significance for differential diagnosis of breast cancer. The golden standard of diagnosis for this disease is pathological examination.^[23]^

Radical resection is the main treatment for this disease.^[24]^ The patient in this case underwent ipsilateral axillary lymph nodes clearance after extensive resection of the tumor. The margin of the lesion and the status of metastasis in axillary lymph nodes were determined by rapid frozen pathological examinations during the operation, to diminish the operational region as much as possible while completely removing the lesion. Postoperative adjuvant therapy has not been standardized. Chemotherapy, radiotherapy, endocrine therapy or target drug therapy should be selected wisely according to the stage of the disease, the immunohistochemical examination results and the metastatic status of lymph nodes.^[25]^ Since the occurrence of this disease is extremely rare, there is currently no treatment guidelines and the large-scale randomized controlled clinical study has not been performed. Under such circumstances, the treatment strategy of accessary breast cancer is always referred to the treatment of breast cancer. In addition, according to the existing relevant literatures and our clinical experience, we believe that prophylactic resection of the accessory mammary glands which have no physiological functions should be performed in patients, regardless of the gender and presentation of symptoms, especially in patients over the age of 30 to 35 years old, as a preventative method of the occurrence of accessory breast cancer. Nevertheless, our clinical understanding of this disease is still insufficient, thus further research and exploration are still of need for clinicians.

## Author contributions

**Data curation:** Minglei Bi.

**Investigation:** Minglei Bi.

**Writing – original draft:** Minglei Bi, Yipeng Su, Pengfei Sun.

**Writing – review and editing:** Minglei Bi, Danyi Li, Pengfei Sun, Yan Gao.

## References

[1] Khanna S Mishra SP Kumar S Carcinoma in accessory axillary breast. BMJ Case Rep 2015;bcr2015210944 doi: 10.1136/bcr-2015-210944. 10.1136/bcr-2015-210944 PMC4533617 26260957

[2] Gutermuth J Audring H Voit C Primary carcinoma of ectopic axillary breast tissue. J Eur Acad Dermatol Venereol 2006;20:217–21. 16441639 10.1111/j.1468-3083.2005.01362.x

[3] Fentiman IS Fourquet A Hortobagyi GN Male breast cancer. Lancet 2006;367:595–604. 16488803 10.1016/S0140-6736(06)68226-3

[4] Bi L Li J Shi Z Male accessory breast cancer successfully treated with endocrine therapy: a case report. Oncol Lett 2015;10:2495–8. 26622878 10.3892/ol.2015.3602 PMC4580083

[5] Zhong GB Ye XQ Liu JL Male accessory breast cancer on the abdominal wall: a case report and literature review. Oncology Targets Ther 2018;11:6625–31. 10.2147/OTT.S184185 PMC6188187 30349296

[6] Niu Q Jiang X Li Q Texture features and pharmacokinetic parameters in differentiating benign and malignant breast lesions by dynamic contrast enhanced magnetic resonance imaging. Oncol Lett 2018;16:4607–13. 30214595 10.3892/ol.2018.9196 PMC6126147

[7] Hao JY Yang CC Liu FF Accessory breast cancer occurring concurrently with bilateral primary invasive breast carcinomas: a report of two cases and literature review. Cancer Biol Med 2012;9:197–201. 23691479 10.7497/j.issn.2095-3941.2012.03.008 PMC3643663

[8] Amsler E Sigal-Zafrani B Marinho E Ectopic breast cancer of the axilla. Ann Dermatol Venereol 2002;129:1389–91. 12536178

[9] Chen Y Fu YB Xu XF Lymphadenitis associated with cat-scratch disease simulating a neoplasm: imaging findings with histopathological associations. Oncol Lett 2018;15:195–204. 29399138 10.3892/ol.2017.7311 PMC5766074

[10] Wang M Liu Y Li D Endobronchial fibroma in a pneumoconiosis patient with a history of tuberculosis: a case report and literature review. Oncol Lett 2016;12:1041–5. 27446391 10.3892/ol.2016.4726 PMC4950651

[11] Zhang D Li X Xiong H Tuberculosis of the parotid lymph nodes: clinical and imaging features. Infect Drug Resist 2018;11:1795–805. 30349336 10.2147/IDR.S164993 PMC6188200

[12] Hu X Zhou X Yang H Axillary ultrasound and fine needle aspiration biopsy in the preoperative diagnosis of axillary metastases in early-stage breast cancer. Oncol Lett 2018;15:8477–83. 29805585 10.3892/ol.2018.8445 PMC5958674

[13] De Alcantara Filho PR Curi C Guatelli CS Intramammary sentinel lymph node with capsular extravasation in breast cancer. Ann Surg Treat Res 2017;92:376–9. 28480185 10.4174/astr.2017.92.5.376 PMC5416920

[14] Soriano-Maldonado A Carrera-Ruiz Á Díez-Fernández DM Effects of a 12-week resistance and aerobic exercise program on muscular strength and quality of life in breast cancer survivors: study protocol for the EFICAN randomized controlled trial. Medicine (Baltimore) 2019;98:e17625. 31689771 10.1097/MD.0000000000017625 PMC6946307

[15] Ach T Ziemer M Dawczynski J Differential expression of tetraspanin CD9 in basal cell and squamous cell carcinomas of the skin and actinic keratosis. Oncol Lett 2010;1:37–40. 22966252 10.3892/ol_00000006 PMC3436406

[16] Suzuki H Hashimoto A Saito R A case of primary cutaneous basal cell carcinosarcoma. Case Rep Dermatol 2018;10:208–15. 30283313 10.1159/000492525 PMC6167722

[17] Chand P Kumar A Singh P Unusual presentation of ulcerative postauricular swelling as sebaceous cell carcinoma. Niger J Surg 2016;22:127–9. 27843279 10.4103/1117-6806.169819 PMC5013740

[18] Rastrelli M Ferrazzi B Cavallin F Prognostic factors in merkel cell carcinoma: a retrospective single-center study in 90 patients. Cancers (Basel) 2018;10:350 doi:10.3390/cancers10100350. 10.3390/cancers10100350 PMC6210570 30249978

[19] Tarapore E Atwood SX Defining the genetics of basosquamous carcinoma. J Invest Dermatol 2019;139:2258–60. 31648686 10.1016/j.jid.2019.04.011

[20] Spallone G Sollena P Ventura A Efficacy and safety of Vismodegib treatment in patients with advanced basal cell carcinoma and multiple comorbidities. Dermatol Ther 2019;32:e13108. 31606940 10.1111/dth.13108

[21] Sano D Fujisawa T Tokuhisa M Real-world treatment outcomes of the EXTREME regimen as first-line therapy for recurrent/metastatic squamous cell carcinoma of the head and neck: a multi-center retrospective cohort study in Japan. Anticancer Res 2019;39:6819–27. 31810948 10.21873/anticanres.13898

[22] Ponti G Manfredini M Tomasi A Muir-Torre syndrome and founder mismatch repair gene mutations: a long gone historical genetic challenge. Gene 2016;589:127–32. 26143115 10.1016/j.gene.2015.06.078

[23] Thorne AL Jackson A Yiangou C The use of sentinel node bniopsy in the treatment of cancer of an accessory breast. Breast 2003;12:153–5. 14659346 10.1016/s0960-9776(02)00266-7

[24] Lombardi A Pastore E Maggi S Positive margins (R1) risk factors in breast cancer conservative surgery. Breast Cancer (Dove Med Press) 2019;11:243–8. 31440079 10.2147/BCTT.S210788 PMC6668245

[25] SH Sringeri RR Chandra PS Role of plasma D-dimer levels in breast cancer patients and its correlation with clinical and histopathological stage. Indian J Surg Oncol 2018;9:307–11. 30287988 10.1007/s13193-017-0682-x PMC6154371

